# Sexual Dimorphism of Immune Responses: A New Perspective in Cancer Immunotherapy

**DOI:** 10.3389/fimmu.2018.00552

**Published:** 2018-03-21

**Authors:** Imerio Capone, Paolo Marchetti, Paolo Antonio Ascierto, Walter Malorni, Lucia Gabriele

**Affiliations:** ^1^Department of Oncology and Molecular Medicine, Istituto Superiore di Sanità, Rome, Italy; ^2^Department of Oncology, Sapienza University of Rome, Sant’Andrea Hospital, Rome, Italy; ^3^Unit of Melanoma, Cancer Immunotherapy and Innovative Therapy, Istituto Nazionale Tumori Fondazione G. Pascale (IRCCS), Naples, Italy; ^4^Center for Gender-Specific Medicine, Istituto Superiore di Sanità, Rome, Italy

**Keywords:** cancer, immunotherapy, sexual dimorphism, interferon, immune response, immune checkpoint inhibitors, therapeutic vaccines

## Abstract

Nowadays, several types of tumors can benefit from the new frontier of immunotherapy, due to the recent increasing knowledge of the role of the immune system in cancer control. Among the new therapeutic strategies, there is the immune checkpoint blockade (ICB), able to restore an efficacious antitumor immunity and significantly prolong the overall survival (OS) of patients with advanced tumors such as melanoma and non-small cell lung cancer (NSCLC). Despite the impressive efficacy of these agents in some patients, treatment failure and resistance are frequently observed. In this regard, the signaling governed by IFN type I (IFN-I) has emerged as pivotal in orchestrating host defense. This pathway displays different activation between sexes, thus potentially contributing to sexual dimorphic differences in the immune responses to immunotherapy. This perspective article aims to critically consider the immune signals, with particular attention to IFN-I, that may differently affect female and male antitumor responses upon immunotherapy.

In the last decade the increasing knowledge of the role of the immune system in cancer control has led to the development of the immune checkpoint inhibitors (ICIs), that targeting proteins acting as negative regulators of T-cell activation, reverse the tumor-induced immune tolerance.

The main actors of this scene are two key molecules called cytotoxic T-lymphocyte-associated protein 4 (CTLA-4) and programmed cell death receptor 1 (PD-1) (1). CTLA-4 binds B7 receptors on antigen-presenting cells (APCs), thus blocking T-cell activation. Likewise, PD-1 expressed on activated T lymphocytes, upon interaction with its ligands PD-L1 and PD-L2 on APCs or tumor cells, limits their activity delivering negative signals. PD-1 is also expressed by regulatory T cells (Tregs), enhancing their function. The clinical use of ICIs has extraordinarily increased overall survival (OS) in patients with cancer, suggesting that targeting the immune checkpoint blockade (ICB) is a privileged strategy for fighting cancer (2, 3). A crucial part of this game is played by tumor-infiltrating T cells (4) and in many cases, the effectiveness of ICIs is limited by the lack of adequate antitumor immunity in the tumor microenvironment (TME) (5). To overcome this limitation, new combination therapies are being investigated (6). In this landscape, a new perspective for improving the efficacy of immunotherapy is to take into account sexually dimorphic differences of immune responses (7).

## Components of Antitumor Immunity Critical for Immunotherapy

In the era of ICIs, T-cell immunity remains central for tumor regression (8). This is far from being simple and occurs only when diverse elements coexist either during cancer immunosurveillance or immunotherapeutic treatments (9). Cancer cells carry tumor antigens, and in particular neoantigens, that are the most capable of inducing an effective T-cell immunity. These antigens, mainly present in tumors with high mutational rate, generate T-cell responses upon processing and presention by DCs. Accordingly, CD8+ T cells infiltrates at high frequency these tumors and this associates with a patient survival advantage (10). Nevertheless, in specific conditions tumor-infiltrating DCs lack their immunostimulatory function and acquire immunosuppressive activity (11). This relies on different phenotypes and functions of DC subsets that provide additional variability to the onset of antitumor responses. Within human tumors, rare BDCA3+ DCs were found extremely competent in processing and cross-presenting antigens, driving the expansion of tumor-specific cytotoxic T cells (CTLs) (12). These DCs are the equivalent of the mouse CD103+ DCs, depending uniquely on DC-lineage committing transcription factors such as interferon regulatory factor 8 (IRF-8) (13). Tumor-infiltrating CD103+/BDCA3+ DCs represent privileged players for responses upon ICB (14, 15). However, in TME the most abundant DCs show immunosuppressive activity, including plasmacytoid DC (pDCs). Activation by toll-like receptor 7 (TLR7) ligand reverses pDC immunosuppressive function to such an extent that their administration to melanoma patients induces tumor-specific CD4+ and CD8+ T-cell responses (16, 17). PDCs are the main producers of IFN-α (18), a well-known cytokine linking innate and adaptive immunity and endowed with potent direct and immunomediated antitumor effects (19). Endogenous IFN-I is required to initiate antitumor response in the elimination phase of cancer immunoediting (20), and IFN-I production is also essential for tumor rejection by DC-stimulated activated T cells (21). IFN-I specifically improves the ability of CD8a+ CD103+ DCs to cross-prime tumor specific CD8+ T cells (22–24). However, although IFN-I is crucial for the outcome of ICB, its activation may produce an opposite role in a time-dependent manner (25). In melanoma, while early IFN-I activation correlates with an effective PD-1 blockade (5, 26), prolonged IFN-I signaling seems to favor resistance (27). This apparent discrepancy might not be surprising since, during persistent viral infections, IFN-I displays protective effects in early stages and becomes detrimental upon continued signaling activation in the chronic phase (28). Interestingly, CML patients in remission who stopped IFN-α treatment because of good clinical response developed higher protective T-cell memory response than patients who continued therapy (29). In TME, while IFN-I signaling is critical for survival and full activation of CD8+ T cells, the continuous generation of terminally differentiated CD8+ T cells might determine the failure of ICB by reducing progenitors able to respond (30). Thus, the generation of exhausted CD8+ T cells may be the results of complex immunosuppressive interactions including prolonged IFN-I signaling (31). Indeed, IFN-I is critical for the induction of immune checkpoints or coinhibitory receptors, such as PD-1 and T-cell immunoglobulin- and mucin-containing molecule-3 (Tim-3) (32, 33), whose persistent overexpression characterizes exhausted T cells within TME (34). Moreover, acute and chronic IFN-I expression impacts Tregs differently. While IFN-α abrogates the suppressive activity of CD4+ CD25+ Foxp3+ Tregs, thus hampering tumor evasion (35, 36), IFN-I signal blockade boosts the ICB-induced antitumor response by favoring the effector T cells to Tregs ratio (37). Of interest, Treg-mediated immune tolerance may occur through the control of IFN-I on indoleamine 2,3-dioxygenase (IDO) (38, 39), being melanoma peritumoral IDO expression by pDCs an early marker of resistance (40). Noteworthy, both Tregs and IFN-I also suppress the function of natural killer (NK) cells whose antigen-independent cytotoxicity is the second effector mechanism responsible for an efficacious antitumor response (41, 42). Altogether, these immune components build a functional framework for innate and acquired resistance to PD-1 blockade (43), where the balance between inflammation and suppression determined by the fine-tune regulation of IFN-I is crucial. This central achievement occurs through the modulation of different transcriptional programs involving the Janus kinase (JAK)-signal transducer and activator of transcription (STAT) and IFN regulatory factors (IRFs) families, as well as proteins of the phosphatidyl inositide3-kinase (PI3K) and mitogen-activated protein kinases (MAPK) pathways (44). Moreover, epigenetic signals, such as histone modification, DNA methylation, and microRNAs (miRs), represent a second key layer of regulation (45). Indeed, exhausted CD8+ T cells within tumors display a distinct epigenetic profile and the limited remodeling may represent a pivotal component of resistance to PD-L1 blockade (46). Likewise, miRs modulate the expression of PD-L1 potentially accounting for innate resistance (47). Finally, it is worthy to mention drug toxicity as one of the major cause of reduced dosage, delayed drug administration and therapy discontinuation (48). Immune-related adverse events (irAEs), such as the loss of the protective function of intestinal barriers and changes occurring in the microbiota composition, represent the most frequent ICI-associated toxicities to which dysregulated activation of the IFN-I signaling may contribute (49, 50).

## Sexual Dimorphism of the Immune Components of Host Response to Immunotherapy

**Figure 1:**
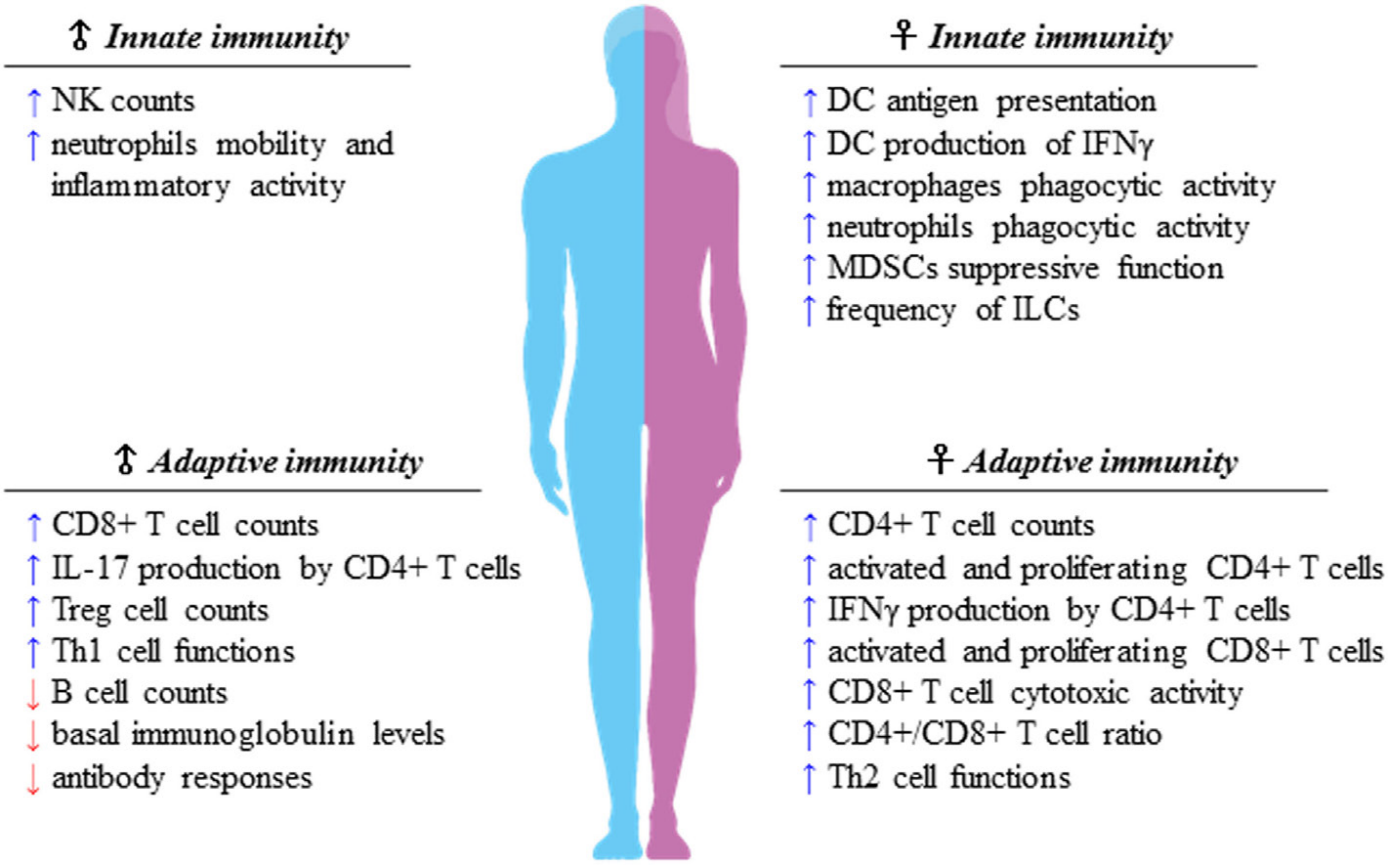
Sexual dimorphism of the immune responses. Immune components of both innate and adaptive immunity are differently regulated in females and males. Apparently, females display higher capability of mounting type-2 versus type-1 immune responses, whereas males seem to prefer type-1 immune responses, of which many traits are still unclear. As a matter of fact, the difference of the strength of type-1 immunity between sexes is smaller than that of type 2, preserving the onset of female inflammatory cell-mediated immune responses.

**Figure 2:**
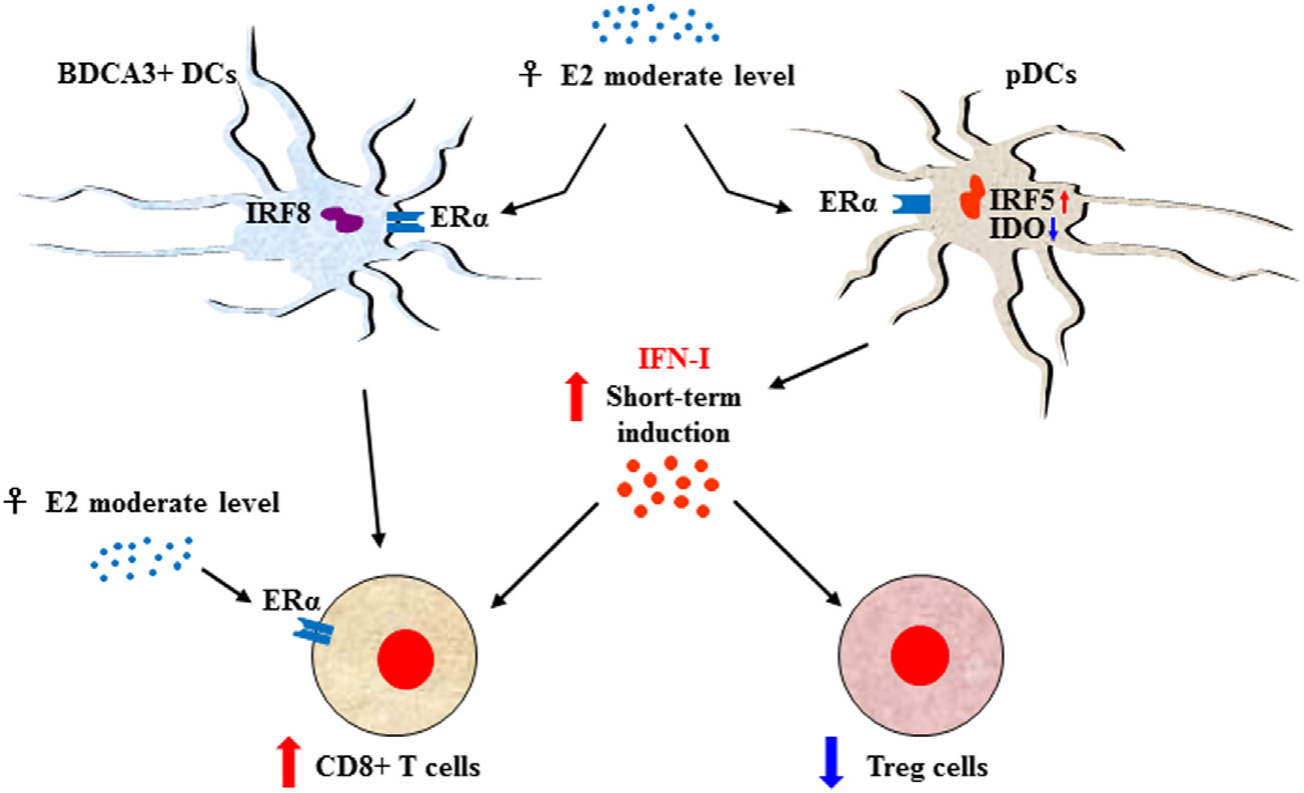
Sex-biased regulation of T-cell response through E2-induced IFN type I (IFN-I) production. The E2–ERα axis controls the functional responses of diverse DC subsets along with IFN-I production. In females, upon 17β-estradiol (E2) stimulation plasmacytoid DC (pDCs) express enhanced interferon regulatory factor 5 (IRF-5) and reduced indoleamine 2,3-dioxygenase (IDO) leading to transient production of high levels of IFN-α that, in turn, stimulate CD8+ T-cell activity and downregulate regulatory T (Treg) cells. CD8+ T cell can also be stimulated by activation of BDCA3+ DCs, whose activation is interferon regulatory factor 8 (IRF-8) dependent. Moreover, the activity of CD8+ cells is directly modulated by the E2–ERα axis in a hormone dosage-dependent manner. In this context, IFN-I maybe the signal which, in some conditions such as the onset of the antitumor response restored by immune checkpoint inhibitor, drives a more powerful inflammatory cell-mediated immune response in female.

Sexual dimorphism of the immune functions is a crucial element that has so far been largely ignored in the field of immunotherapy (51). These differences affect both innate and adaptive immune responses, leading to a considerable functional diversity between females and males (7) (Figure 1). Sex variations include the number and activity of cells as well as intracellular and extracellular signals orchestrating the two branches of immunity. In the innate context, females own APCs that perform antigen presentation more vigorously, have neutrophils and macrophages endowed with higher phagocytic activity, and show a higher frequency of both progenitors and mature group 2 innate lymphoid cells (ILCs), key regulators of type-2 inflammatory responses (52). On the contrary, males exhibit enhanced numbers of NK cells (7). In the adaptive context, females exhibit higher CD4+ T cell counts associated with an increased CD4+/CD8+ T-cell ratio, along with Th2 prevalence, and greater proliferation and cytotoxicity of T cells. In contrast, males have higher CD8+ and Treg cell counts associated with Th1 dominance, lower B cell numbers and basal immunoglobulin levels along with weaker antibody responses (53). Both hormonal and genetic differences concur in the sexual dimorphism of the immune system. The 17β-estradiol (E2) –estrogen receptor α (ER) axis is a key regulator of innate immune populations. E2 reduces mobility and inflammatory activity of neutrophils (54), and female mMDSCs seem to be more suppressive than the male counterpart (55). Importantly, the E2–ERα axis exerts a tight control on the functional responses of diverse DC subsets, also by modulating IFN-I production. Moreover, high levels of E2 promote epigenetic changes in DC precursors of females driving DC differentiation and robust IFN-I production (56) (Figure 2). PDCs are the DC with major differences between the sexes. Their activity is driven by TLR7, whose gene located on X chromosome and under the E2–ERα signaling (57, 58) promotes high IFN-α production through enhanced expression of IRF-5 (59) (Figure 2). Nevertheless, the development and function of other DC subsets are also affected by the E2–ERα axis (60). In female mice, CD103+ conventional DCs (cDCs) are represented at very high levels in the cutaneous lymph nodes and CD103− cDCs show high expression of ERα in the lung (61, 62). Furthermore, E2 treatment has been reported to enhance IRF-4+ DC capability to stimulate Th17 in a murine model of HSV-2 infection (63), while it induces IFN-α and IL-6 production as well as CD40, CD86, and MHCII expression in cDCs. In contrast, tolerogenic FOXO3-expressing DCs display lower frequency and reduced function in tumors from females compared with their male counterpart (64). Altogether, these evidences confirm a positive regulatory feedback loop between the E2–ERα and IFN-I signals in regulating the phenotype and function of DC populations (65). DC differentiation and function are also affected by other hormones, such as prolactin, progesterone and glucocorticoids, driving either a pro-inflammatory or a tolerogenic phenotype (66). In this light, the sex-specific components of the innate immunity become important in improving cancer immunotherapy. Many elements of the adaptive immunity are regulated by the E2–ERα axis as well. Low levels of estrogen, as during the luteal phase, favor Th1 polarization of CD4+ T cells associated with increased production of IFN-γ, responsiveness to IL-12 through STAT-4 activation, and T proliferation. Conversely, high levels of estrogens, found in the follicular phase and during pregnancy, sustain Th2-mediated immunity characterized by IRF-1-mediated reduction of IFN-γ, IL-4 induction, and PD-1-overexpressing Tregs associated with reduced Th17 response (67). Nevertheless, estrogen together with other factors may directly stimulate Th17 cells inducing IL-17 production (68). Thus, females exhibit activated and proliferating CD4+ and CD8+ T cells, characterized by preferential production of IFNγ and high-cytotoxicity activity, whereas males exhibit IL-17-producing T cells (69). This underlines how sex hormones become crucial in determining the efficacy of some therapies. B16 melanoma-bearing female mice, more than males, benefit from ICB, partially due to a greater PD-L1 blockade-mediated reduction of Treg function (70).

The molecular point of view of the sexual immune dimorphism is also extremely intriguing. Many key immune-related genes, such as FOXP3 and CD40L, are located on the X chromosome (71, 72), and numerous X-linked genes of T cells carry the estrogen response elements (EREs) in their promoter. Hence, T cells from women may display a sex-biased signature characterized by inflammatory/cytotoxic effector genes such as IFN-γ, granulysin (GNLY), granzyme A (GZMA), RIGI, LTβ, IL12Rβ2, OAS1, IF16, X3CL1, CX3CL2, and the cytokines IL-15 and IL-16 (73). Therefore, the X chromosome may be responsible, at least in part, for the immunological advantage of females, whose signals are potentially more suitable to be activated by immunotherapies. This assumption is confirmed by the finding that the X chromosome is highly enriched in miRs as about 118 are located in this position, whereas only two miRs have been identified on the Y chromosome (74). MiRs sex-specific expression may have an enormous regulatory power on immune responses, through the control of signals and function of specific immune cell populations. For instance, CD4+ T cells from female lupus patients have been found to overexpress 18 X chromosome-linked miRs with respect to the male counterpart (75). The PD-L1 expression itself, directly or through the control on trascription factors, appears to be modulated by several X-linked miRs, including miR-221, miR-222, miR-106b, miR-20b, and miR-513 (47); of interest, the X-linked miR-424 targets both PD-L1 and CD80 regulating concurrently the PD-L1/PD-1 and CD80/CTLA-4 pathways (76). Notably, a close crosstalk has been reported between the E2–ERα axis estrogen activity and epigenetic regulation by X-linked miRs, since molecules such as miR-221 and miR222 bind and regulate the ERα transcript (74).

The impact of sex differences on antitumor immunity is critical. In melanoma patients, partially exhausted cytotoxic CD8+ T lymphocytes (peCTLs), upon increased expression and engagement of CTLA-4 and PD-1, drive the dysregulation of the host response. Therefore, while both sexes with high peCTLs show a similar objective response rate (ORR) following anti-PD-1 monotherapy as well as combination therapy, females with low peCTLs exhibit higher ORR after combination therapy (77). In the sex-biased immune landscape the complex interplay between microbiome, host immune system, and tumor is pivotal (49). The composition of gut microbiome affects responsiveness to ICIs, as melanoma patients with a greater variety of gut bacteria have high frequence of tumor-infiltrating CTLs predicting a better prognosis (78). In a murine melanoma model, the commensal *Bifdobacterium* was shown to improve ICB through DC activation associated with higher tumor-infiltrating CD8+ T cells (79). Also, irAEs, such as diarrhea and colitis due to gut bacteria composition changes and intestinal barrier dysfunction (49), may have a sex-biased occurrence as gut microbiome is under a hormone-dependent control (80).

## The Implementation of Immunotherapy on a Sex-Based Perspective

Vaccines was one of the first cancer immunotherapy approach. Despite most of them elicit antigen-specific immune responses in several clinical settings, they have largely failed in achieving a survival benefit (81), due to both tumor-cell intrinsic and extrinsic factors circumventing immune recognition and creating a suppressive TME (82).

Among therapeutic cancer vaccines, DCs have been regarded as a promising approach (83) and have been tested on wide range of tumor types (84). A comprehensive meta-analysis has demonstrated tumor-specific T-cell response in 77% of prostate and 61% of Renal Cell Carcinoma (RCC) patients (85). Although a correlation between DC-induced antigen-specific immune responses and prolonged patient survival has been confirmed in many studies (86, 87), the ORR does not exceed 15–20% (88). Moreover, most of these studies are phase-I/-II trials, involving few patients, whose immune and clinical responses were not evaluated taking into account patient characteristics, including sex. Future promising areas of investigation will be the use of personalized vaccines targeting neoantigens and their combination with immunomodulatory agents limiting the inhibitory signals in the TME (82, 89). In this context, due also to the hormonal modulation of many immune populations, including DCs (66), it would be extremely beneficial to consider how to take advantage of the sex-specific immune components.

A major paradigm shift in cancer immunotherapy was the use of antibodies targeting the immune-inhibitory pathways to unleash anticancer T-cell responses (90). Ipilimumab, targeting CTLA-4, was the first antibody clinically approved for treating patients with advanced unresectable melanoma (91). Soon after, nivolumab and pembrolizumab were developed to target PD-1. Today, ICIs are in the front line of immunotherapy of various advanced cancers; partial or complete objective responses have been obtained in patients with melanoma (31–44%) (92), NSCLC (20%) (93), and RCC (22–25%) (94). However, while such therapies exhibit clinical efficacy in many patients, the lack of response in a significant fraction of them remains the major concern. In this regard, intratumoral-infiltrating T cells correlate with a favorable outcome in melanoma patients, to the extent that analyses of type, functional orientation, density, and spatial location of these cells have been developed into a predictive immune scoring system (95). Accordingly, ICI efficacy seems to correlate with an ongoing cellular immune response and patients who do not respond to therapy often present metastatic lesions poorly infiltrated with immune cells (96).

Nevetheless, other crucial components concur to ICI treatment failure, including DC breakdown in proper Ag presentation to effector T cells, persistence of exhausted T cells, and lack of T-cell memory in TME (4, 9). Upon therapy, this dysfunctional state can be reversed or not. In this light, IFN-I signaling has been identified as an important factor for both response and resistance to ICI therapy (25). In fact, IFN-I may have immuno-suppressive or stimulatory activities depending on the magnitude, the timing and the duration of the activation of the signaling. Therefore, the activation of the IFN system may be crucial for the initial response to anti-PD-1, potentially favored in females who have an enhanced aptitude to activate this signal. It may, however, be detrimental if prolonged, promoting escape and resistance in patients who first responded (26, 27). In this light, the implementation of ICI therapies should take into account also the timing of the activation of the sex-specific components of the antitumor response.

Recently, a meta-analysis evaluating sex-related differences in the response to ICIs was carried out (97). The study assessed the progression free survival (PFS) and the OS in selected 36 phase II/III clinical trials on patients with solid tumors, including melanoma, NSCLC, RCC, head and neck, and urotheial carcinoma, and treated in the first or second line with ipilimumab and/or anti-PD-1 antibodies. Overall, 3,274 patients, of which 2,007 males (61.3%) and 1,267 females (38.7%), were analyzed. The results showed a better OS associated with ipilimumab in males compared with females. Not statistically significant results were observed with anti-PD-1 neither for OS nor for PFS. It should be emphasized that this analysis presents some weaknesses and limitations depending on the heterogeneity of the trials, the different cancer types considered, the variability of treatment regimens including patients treated or not with previous therapies, and the absence of information about hormonal status and on PD-L1 expression according to sex.

## Conclusion

Research in cancer therapeutics has largely focused on two distinct approaches. One is based on the characterization of mutations causing tumorigenesis pivotal for the use of drugs targeting altered proteins in cancer cells; the other treats cancer “indirectly,” exploiting the activation of the immune system to generate an antitumor immunity. While the first approach elicits impressive, but often not durable, tumor responses, the second accomplishes durable clinical responses but only in a subset of patients, a fraction of which experiences relapse after initial encouraging responses. Mechanisms of primary or acquired resistance are the major obstacles in both cases. Sex-associated factors correlate with cancer incidence, outcome, and response to therapy, underlining that sex differences are critical in tumor–host interaction. In this framework, sex disparity in immunity have been recently “re-discovered” and IFN-I signal could play a pivotal role in this scenario. Thus, the sexual dimorphism of the immune signals, including the IFN-I ones, may be a new attractive perspective for optimizing immunotherapy. Moreover, this critical challenge could represent a future opportunity to better integrate immunotherapies with other conventional as well as targeted therapies.

## Author Contributions

All authors contributed to the writing and editing of the manuscript.

## Conflict of Interest Statement

The authors declare that the research was conducted in the absence of any commercial or financial relationships that could be construed as a potential conflict of interest.
